# Thermoplasmonic Study of a Triple Band Optical Nanoantenna Strongly Coupled to Mid IR Molecular Mode

**DOI:** 10.1038/srep22227

**Published:** 2016-02-26

**Authors:** Dihan Hasan, Chong Pei Ho, Prakash Pitchappa, Bin Yang, Chunsheng Yang, Chengkuo Lee

**Affiliations:** 1 Department of Electrical & Computer Engineering, National University of Singapore, 4 Engineering Drive 3, 117576, Singapore; 2 Center for Intelligent Sensors and MEMS, National University of Singapore, 4 Engineering Drive 3, Singapore 117576; 3 National Key Laboratory of Science and Technology on Micro/Nano Fabrication, Department of Micro/Nano Electronics, Shanghai Jiao Tong University, Dong Chuan Road 800, 200240 Shanghai, P. R. China; 4 NUS Suzhou Research Institute (NUSRI), Suzhou Industrial Park, Suzhou, P. R. China 215123

## Abstract

We report the first thermal study of a triple band plasmonic nanoantenna strongly coupled to a molecular mode at mid IR wavelength (MW IR). The hybrid plasmonic structure supports three spatially and spectrally variant resonances of which two are magnetic and one is dipolar in nature. A hybridized mode is excited by coupling the structure’s plasmonic mode with the vibrational mode of PMMA at 5.79 μm. Qualitative agreement between the spectral changes in simulation and experiment clearly indicates that resistive heating is the dominant mechanisms behind the intensity changes of the dipolar and magnetic peaks. The study also unveils the thermal insensitivity of the coupled mode intensity as the temperature is increased. We propose a mechanism to reduce the relative intensity change of the coupled mode at elevated temperature by mode detuning and surface current engineering and demonstrate less than 9% intensity variation. Later, we perform a temperature cycling test and investigate into the degradation of the Au-PMMA composite device. The failure condition is identified to be primarily associated with the surface chemistry of the material interface rather than the deformation of the nanopatterns. The study reveals the robustness of the strongly coupled hybridized mode even under multiple cycling.

Thermo-plasmonics is the study of the heating of metallic structures at nanoscale when electromagnetic wave is captured by the plasmonic resonances^1^,^2^. Temperature control at nanoscale is one of the most critical engineering challenges of nanotechnologies. Plasmonic nanoparticles can be of significant engineering merit in this direction for unraveling the complex processes of nanothermodynamics^3^,^4^,^5^. In the last few decades, the field of plasmonics received extensive attention of the researchers because of plasmon’s unprecedented ability to couple free space electromagnetic excitation into nanoscale volume and manipulate light-matter interaction. With the recent advancement of nanotechnology, plasmonics has become the emerging research topic in energy harvesting, telecom and sensing industries^6^,^7^,^8^,^9^,^10^,^11^. The plasmonic approach for probing thermal effect typically involves the observation of optical index variation in the surrounding as a function of temperature based on the far field optical measurement. The non-radiative decay of the plasmonic excitation is associated with the ultra-fast electron-phonon and phonon-phonon coupling in the lattice. The electron gas in a metal nanostructure, upon interaction with the resonant photons, results in a non-equilibrium heating of the structure and produces a localized temperature gradient which can be sufficient for photothermal therapy and gene release^12^,^13^. An accurate electromagnetic modeling of the abovementioned heating process often requires a nonlinear model accounting for the temperature dependence of the metal permittivity and its impact on the stabilization of optical response^14^. In this work, we adopt a rather deterministic approach requiring an external heat source in order to study the role of temperature increase on the optical response of plasmonic device. The proposed device is a triple band resonant Au nanoantenna coupled to the molecular mode of a thin PMMA overlayer. Multi-band structures are potentially feasible for integration of real time plasmonic detection with microfluidics due to their enhanced integral response^15^. On the other hand, the coupled hybridized mode of the Au-PMMA structure merges the broadband plasmonic mode (“bright mode”) with the narrowband absorption line of PMMA (“quasi dark mode”) and can render maximal optical transparency with asymmetric profile^16^. Besides, the complex shaped bow-tie configuration of the nanostructure possess strong lighting rod effect and can be useful for photothermal/thermoptic activation of the Au-PMMA composite photonic device^17^,^18^. One technological challenge for the thermal applications of the composite device is the poor adhesion of the commonly used PMMA resist with Au surface. To promote the adhesion, the PMMA surface is typically thiolated before the gold deposition^19^ However, there has not been any study on the thermal degradation of viscoelastic PMMA thin film directly spin cast on the gold patterns for photonic applications demanding large resonance contrast with asymmetric profile. Thermal degradation can be a major concern especially for the reliability of thermo-responsive plasmonic devices over time mostly due to the mismatch of thermal expansion coefficient (TEC), inter-diffusion (1.1 e−18 m^2^/s) and weak inter-layer adhesion^20^

In particular, dipolar strength of the molecular vibrational mode can decline at elevated temperature causing homogeneous broadening of the absorption line^21^. This work studies the reliability of the Au-PMMA composite device over multiple thermal cycles within a moderate temperature range (25–125 deg.) and links the failure condition with the Au-PMMA and SiO_2_-PMMA surface chemistry rather than any structural deformation of the nanopatterns. More importantly, here we explore the robustness of the strongly coupled hybridized mode against thermal cycling and propose a mechanism to reduce the relative intensity change and enhance the light-matter interaction at elevated temperature.

## Design details, fabrication and characterization

### Design

**Figure 1:**
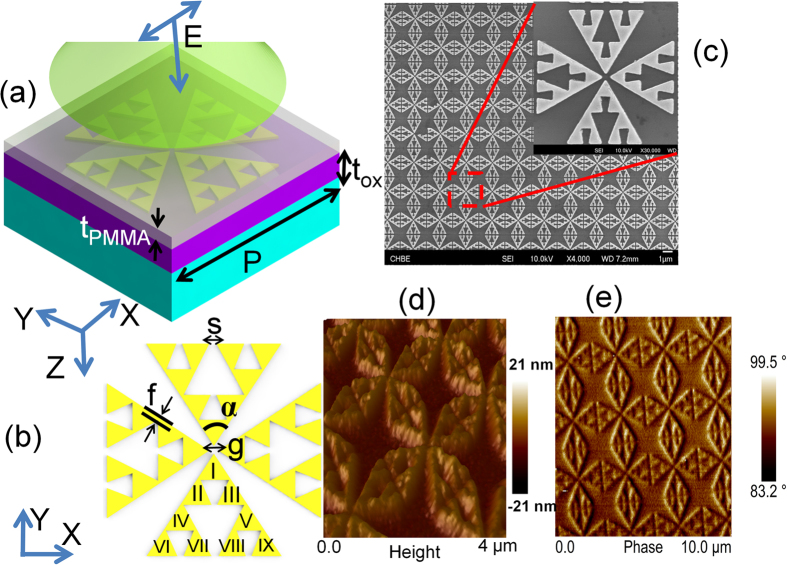
(**a**) 3D schematic of the multi-band resonant plasmonic nanoantenna structure overlaid with 110 nm thick PMMA film (**b**) 2D layout of the structure (**c**) FESEM image of the nanoantenna array (inset: zoomed in view of the nanoantenna unit cell) (**d**) AFM height profile (**e**) AFM phase image of the pattern overlaid in PMMA thin film. The substrate is Si coated with 1 μm thick (t_ox_) thermal oxide.

Figure 1(a) shows the 3D schematic of the nanoantenna structure overlaid in the thin PMMA film. The thickness of the film (t_PMMA_) and the periodicity of the array (P) are fixed at 110 nm and 2.77 μm, respectively. Figure 1(b) shows the 2D layout of the structure. The gap size (g) and the vertex angle (α) are fixed at 100 nm and 60 deg., respectively. The polarization independent configuration is obtained by placing four sets of modified bow-tie triangles in close proximity of nanoscale to each other. The modification includes etching plasmonic voids following the algorithm of a 2^nd^ order *Sierpiński* gasket^22^ in order to pursue three specific objectives: (i) to lessen the intrinsic damping effect on the quality factor by reducing metal surface area (ii) to excite both dipolar and magnetic resonance on the same platform and (iii) to reduce the impact of thermal expansion coefficient (TEC) mismatch between the pattern and the substrate and to increase the spatial field coupling between the pattern and the overlayer (superstrate). Please note that by integrating the voids into a bow-tie arm triangle, the host triangle can be assumed to be consist of 9 quasi self-symmetric radiators as numbered from I to IX. Previously, the bow-tie shaped nano-structure has been extensively engineered to achieve multi-spectral characteristics and ultra-high field enhancement^23^,^24^. The current version of this work is deliberately structured to obtain multiple resonances across the broad spectrum of mid IR wavelengths of which two are magnetic and one is dipolar in nature. We provide the parameter: offset (f) not only to avoid the geometric singularities in simulation and fabrication but also to exploit the strong impact of lightning rod derived surface currents on resonance properties^25^. We further provide the split gap (s) in order to achieve a dramatic enhancement of resonance contrast and optical magnetism in mid IR. The values of f and s are fixed at 70 nm and 100 nm, respectively unless otherwise stated.

### Fabrication

The structures are written by a high resolution 100 KV ELS-7000 Electron Beam Lithography equipment using raster scanning. The e-beam current is maintained at 200 pA. No proximity correction or ITO charge compensation layer has been considered for the current batch of chips. Thus, the issue of back scattering induced corner roundedness may be crucial in some cases. The development time is controlled at 70 s in 1:3 MIBK:IPA solution. Finally, 5 nm of Cr layer followed by 35 nm of gold layer has been deposited at a rate of 1 angstrom/sec by the Denton Explorer e-beam evaporator while maintaining the vacuum level at 5e-7 torr. The metal lift off has been performed by soaking the samples in acetone solution overnight followed by a 5 minute long ultrasonic agitation. Figure 1(c) shows the FESEM picture of the nanoantenna array along with the zoomed in image of the unit cell. The sample is spin coated by a 110 nm thin PMMA film in order to excite the hybridized mode. Figure 1(d) provides the height AFM of the spin coated sample. To assess the conformity of the coating, the phase AFM is also provided in Fig. 1(e). A phase angle deviation of 16.3 deg. is observed implying the high degree of conformity of the coating over the nanopatterned structure. The phase distribution will be also useful to study the thermally induced change of topography later on. The AFM images have been captured by the Bruker AFM with a Si tip of 5 nm radius and the post processing is done by NanoScope Analysis.

### Characterization

A three dimensional FDTD solver has been deployed on a 16 core, 2.60 GHz Intel Xeon ES-2670 (128 GB memory) processor to simulate the reflection and transmission spectrum at near field under y-polarized light. The near field transmittance has been captured by a transmission monitor placed 15 μm away from the metal patterns. The simulation region has been terminated by periodic boundary condition along x and y direction and PML along z direction. A spatial resolution of dx = 1 nm, dy = 1 nm and dz = 1 nm is chosen for accurate calculation. Optical properties of gold have been extracted from Palik and the refractive indices of Si and SiO_2_ have been fixed at 3.5 and 1.45, respectively. The normalized transmission and reflection spectra have been obtained by a broadband Agilent Fourier Transform Infrared (FTIR) Spectroscopy. The area of the aperture is considered to be 100 μm by 100 μm. The reflectance of the devices is normalized with respect to that of a smooth gold surface and transmittance is normalized with respect to the free space transmission of light. The size of the aperture was adjusted carefully to match with the size of the each square pattern, therefore eliminating the background reflection. The sampling resolution is maintained at 4 cm^−1^ to minimize the presence of water absorption peaks although CO_2_ peak at 4.26 μm can appear in the spectrum depending on the ambient concentration. No polarizer has been used in order to perform polarization independent measurement. The mirror repetition rate is fixed at 40 KHz and the number of scans is maintained to be 64.

The sample is placed on a heating stage, which is capable of heating up to 450 °C. The temperature within the stage is controlled by a temperature controller with a variation of less than 5%. The sample chamber is properly insulated from the ambience. The measurement is taken 5 minutes after the stable reading of a particular temperature is reached. In the current setup, only reflection signal can be captured while the heating stage is incorporated (Figure S1, supplementary information).

## Results and Analysis

### Optical Modeling and Design Verification

**Figure 2:**
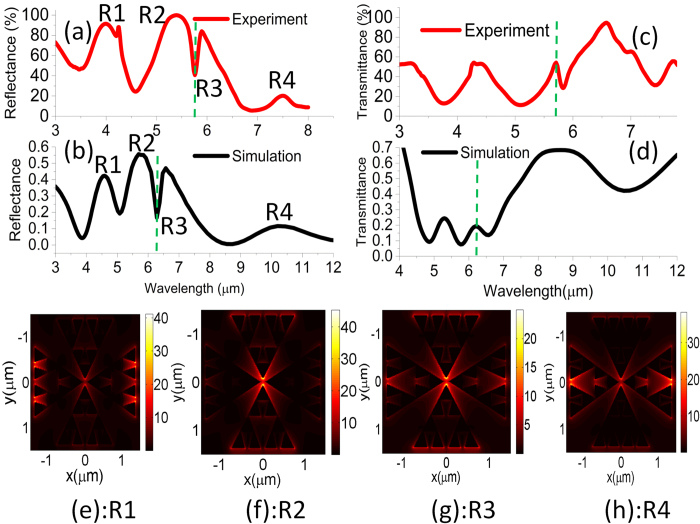
Reflectance spectrum (**a**) experiment (**b**) simulation. Transmittance spectrum (**c**) experiment (**d**) simulation. E-field distribution at resonance location (**e**) R1 (**f**) R2 (**g**) R3 and (**h**) R4. Here, R1 and R4 have the electric field distributions across the horizontal triangles (along x-axis) which look similar to the electrically excited magnetic mode whereas R2 has purely dipolar electric field distribution across the vertical triangle (along y-axis). R3 implies the coupled mode in the vicinity of the R2 and PMMA absorption.

Figure 2(a–d) show the normalized reflection and transmission profile at four different regimes R1, R2, R3 and R4. The spectral positions of the R1, R2, R3 and R4 regimes in experiment are 4 μm, 5.25 μm, 5.73 μm and 7.5 μm, respectively. Generally speaking, the presence of the PMMA superstrate can strongly influence the dipolar moment of the structure due to the local refractive index dependence as speculated by the equation 
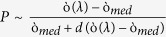
, where P is the dipolar moment, 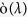 is the wavelength dependent metal permitvitty, 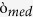 is the permitivitty of the surrounding and *d* is the depolarization factor^26^. According to the effective medium theory (EMT), PMMA superstrate with refractive index 1.4~1.6 will increase the averaged surrounding index beyond 1^27^. Consequently, we observe a strong red-shift of all the resonance peaks when the sample is conformally coated by the PMMA thin film. The optical index of PMMA at mid IR is modeled using the following Lorentzian equation^28^,


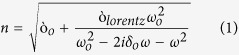


where 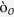 and 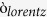
is set at 1.00 and 0.04, respectively and
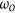
corresponds to the vibrational mode (carbonyl (C=O) stretch) wavelength which is 5.79 μm. The line width of the mode (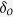) is set at 8e11 rad/s. The spectra in Fig. 2 elucidate a strong agreement between the simulation and experiment except for the mismatch in the spectral position. It can be understood that the reflection peaks as well as the transmission dips at R1, R2 and R4 regimes are blue shifted in the experiment compared to the simulation results for the similar dimensions. The primary reason behind such shift is the corner roundedness that mainly occurs due to the backscattering of e-beam. A careful inspection into the nanometric details reveals the strong presence of corner roundedness of different radii (Figure S2, supplementary information). Such imperfection can cause the spreading of Columbic charges and yield strong blue-shift of resonance in the far field by increasing the restoring force^29^,^30^. Aside from the roundedness effect as previously mentioned, such blue-shift is also a byproduct of the plasmon damping in the far field when the near field coupling is potentially strong^31^,^32^,^33^ and can be verified by simulating the structures replacing the real metal by perfect electric conductor (PEC). Due to the routinely observed shift in the experimentation, it also becomes a challenging task to match the simulated line shape of the hybridized mode with the experimental line shape at the R3 region. To circumvent this, we sweep the vibrational wavelength in the actual Lorentzian model of PMMA and mimic the scenario of the experiment. The results show how the reflection “dip” with the asymmetric line shape begins to appear as the vibrational wavelength reaches 6.35 μm (Figure S3, supplementary information). The variation of the resonance intensity between experiment and simulation is also observed which is due to the background reflection from the oxide coated substrate and dispersion across the large spectrum (Figure S4, supplementary information). However, it is reasonable to argue that such unwanted shift and intensity variation will not affect the fundamentals of the thermal study carried on the device and hence we focus on any relative change of intensity and spectral position as the temperature is increased.

### Thermal effect on the PMMA thin film and the Overlaid Device

**Figure 3:**
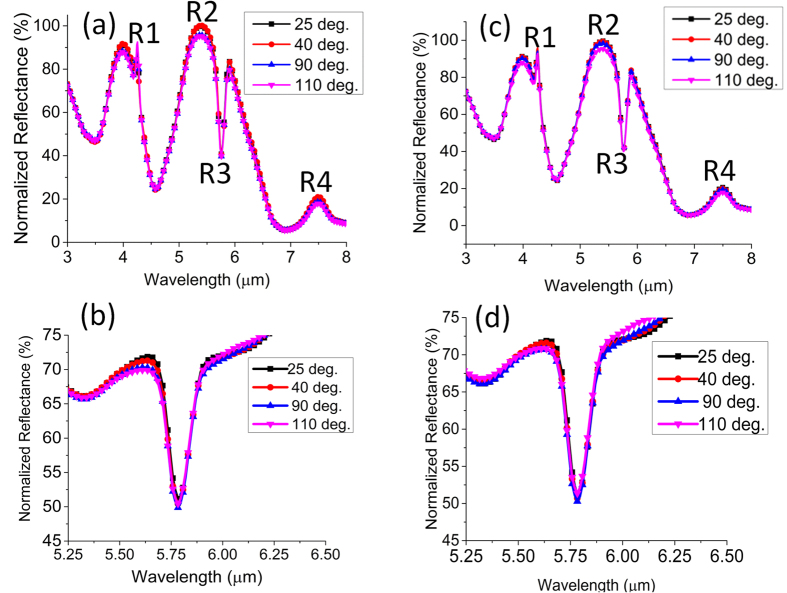
Reflectance spectrum of (**a**) the device (**b**) 110 nm PMMA film as a function of temperature in the first cycle and in the second cycle (**c**) device (**d**) 110 nm PMMA film. The sharp kink around the R1 region is the CO_2_ peak.

**Figure 4:**
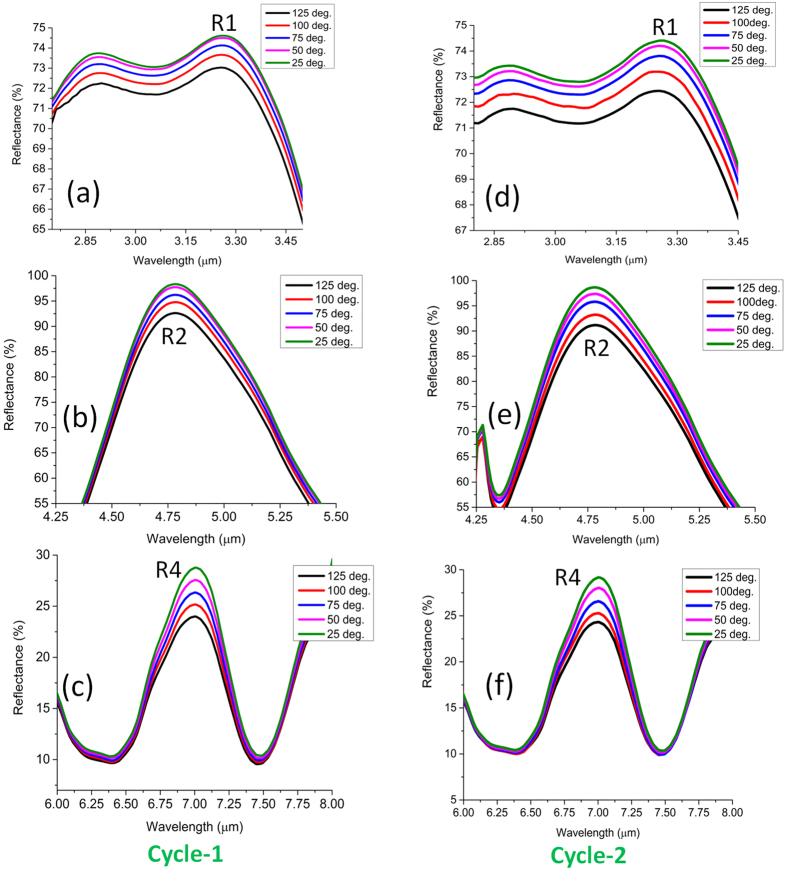
Variation of reflectance of the device without the PMMA overlayer in the first cycle at (**a**) R1 (**b**) R2 (**c**) R4 region and in the second cycle at (**a**) R1 (**b**) R2 (**c**) R4 region.

Figure 3(a) shows the experimentally observed reflection spectrum of the device at four different temperatures: 25 deg., 40 deg., 90 deg. and 110 deg in the first cycle. The upper limit of the temperature is restricted by the glass transition temperature (T_g_) of PMMA organic substance although for thin film, it can increase on SiO_2_ surface and decrease on Au surface^34^. The uneven distribution of temperature values is chosen in order to clearly illustrate the temperature dependence of the spectral features of the device. We calculate an intensity decrease of ~3–5% of the reflection peaks at the R1, R2 and R4 regions as the temperature is increased from 25 deg. to 110 deg. Interestingly, no significant change of spectral positions (R1, R2, R4) of the features is observed. On the contrary, almost no change of intensity (0.5%) of the reflection dip of the coupled mode (R3) is observed for the similar variation of temperature. Please note that the reflection dip corresponds to the transmission peak of the EIT(electromagnetically induced transparency)-like transmission window determined by the two new Eigen states originated from the coupling of the “bright mode” and “quasi dark mode” (Figure S5, supplementary information)^35^. The slight discrepancy between the reflection dip and the transparency peak can be attributed to the mismatch of the incidence angle in the reflection mode and the transmission mode of the FTIR. The thermal experiment is performed again on the PMMA thin film coated on the 1 μm thick oxide atop Si substrate under 45 deg. incidence in the reflection mode. Under homogeneous premise, the temperature dependence of the line width δ (T) of vibrational absorption follows a power law 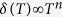, where the exponent n is greater than 1^36^. After the necessary base line correction, line width broadening is indeed observed for the thin film in Fig. 3(b). In particular, at least 2% variation of intensity is observed at the overshoot region on the blue side of the dip. The overshoot develops mainly due to the complex interplay of the real part and the imaginary part of the PMMA refractive index around the transition zone. Please note that the thermo-optic coefficient (dn/dT) of PMMA is −1.05 e10^−4^ K^−1^ implying a refractive index decrease of 0.0105 due to a 100 deg. temperature increase^37^. Such small amount of change will induce a reflectivity change of 0.1338% according to the Fresnel equation. In fact, no change of the spectral features (R1, R2, R4) of the device is observed for the similar order of refractive index change (Figure S6, supplementary information). Thus the intensity variation at the overshoot regime is a strong indication of the line width broadening as predicted by the power law. Nonetheless, the degree of asymmetry of the reflection dip obtained from the device is more significant and the resonance contrast is at least 3 times larger as compared to the thin film. It is important to note here that thermal oxidation and depolymerization of PMMA occurs at much higher temperature beyond the range considered in this work^38^. Interestingly, the temperature dependent spectral change of the device observed in the first cycle is repeated in the second cycle as shown in Fig. 3(b) without exhibiting any major deviation except the minor change in the amount of variation at the R1, R2 and R4 regions. The reflectance is still found to decrease at those regions as the temperature is increased. Such reversible nature of the temperature dependence is a strong indication of the dominant influence of the material properties. In fact, the dipolar intensity of the C=O bond can be a strong function of temperature. Although certain conformational change and thermal stress that may build up in the bare PMMA thin film after the first cycle, can affect the reversible change, the intensity variation in the overshoot region on the blue side implies the line width broadening to some extent even in the second cycle^39^. The comparable

average intensity distribution in the second cycle also nullifies the dominant influence of water content, if any, in the film. Next, another sample of nanoantenna is experimented without the PMMA overlayer in order to confirm the role of dielectric change of metal on the intensity variation at R1, R2 and R4 regions. It is clear from the results rendered in Fig. 4 that the reversible intensity variation of the composite device is mainly attributable to the change of metal permitivitty as the temperature is increased. Detailed modeling results will be provided later in the text to justify such reversible dependence on temperature. Although the reversible nature of the intensity variation strongly indicates the dominant influence of the optical properties over the nanometric structural change at a moderately heated condition, mismatch of mechanical properties among different layers can be potentially vulnerable for the reliability of the device over multiple cycles or in rather harsh conditions. Hence, we also highlight such mismatch and study the spatial distribution of the thermal stress using 3D FEM in the supplementary information (Figure S7)^40^.

### Modeling of thermal effect

As discussed before, the reversible nature of the heating effect is a strong indicator of the metal permitivitty change over multiple cycles. In fact, the decline of the resonance intensity at the R1, R2 and R4 regions is interlinked with the resistive heating caused by the electron-phonon and phonon-phonon coupling. Heuristically, the resistivity change (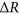) can be written as 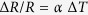, where *α* is the temperature coefficient of resistance. For gold, *α* is equal to 34 × 10 e-4 °C^−1^. Besides, gold is robust against high temperature oxidation with better plasmonic characteristics and particularly suitable for biocompatible applications^41^,^42^. To account for the material change in the optical simulation, we deploy the Drude model of metal permitivitty in a slightly modified form as below^43^,


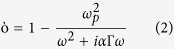


Here, ω_p_ is the plasma frequency, Γ is the Drude damping parameter and α is the factor to quantify the increase in Γ. The nominal values of ω_p_ and Γ are set at 9 ev and 0.07 ev, respectively in the unit of energy. The damping can be splitted into the bulk and surface contributions as below,


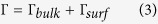


Here, the bulk contribution arises from the electron-electron and electron-phonon interaction and can be expressed as below,


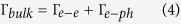


**Figure 5:**
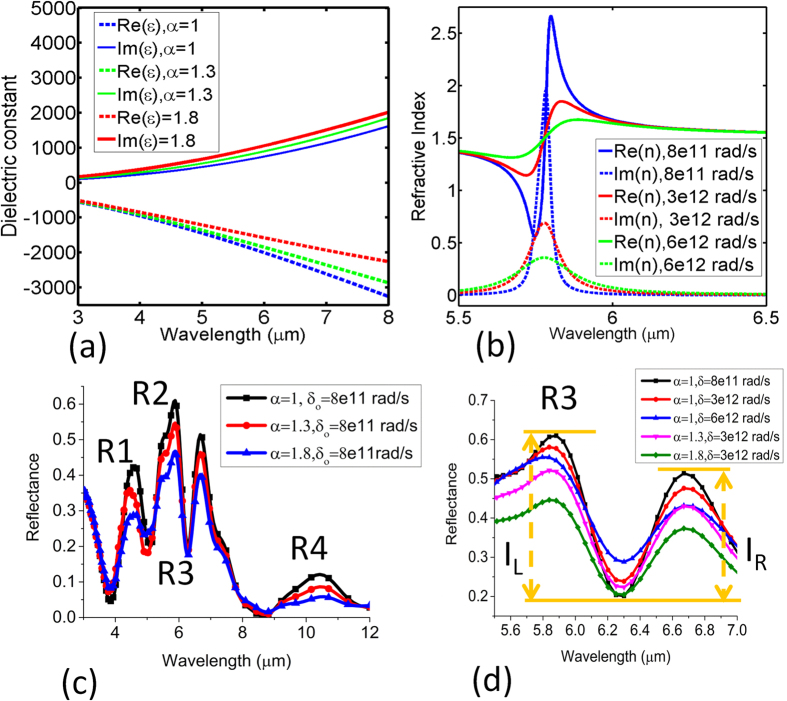
(**a**) Effect of increasing α on the real and imaginary part of metal dielectric constant (**b**) effect of increasing line width on the vibrational resonance of PMMA (**c**) effect of increasing α on the intensities at the important spectral positions of the device at a constant line width of PMMA vibrational mode (**d**) individual effect of increasing vibrational mode line width and α on the reflection dip of the hybridized mode. I_L_ and I_R_ are the resonance contrast of the asymmetric reflection dip on the blue and red side, respectively. The minor kink at the R2 region is due to the fitting error of the modified Drude model by the FDTD package and can be ignored in this work.

Both of these mechanisms are strong functions of temperature and can be accounted with the help of the factor α defined previously^44^. On the other hand, the surface contribution arises due to the limited size of the nanostructure and is a function of the Fermi velocity and reduced mean free path of electrons. In this work, we only increase α to mimic the decrease of DC conductivity at inreased temperature and do not treat the bulk and surface contribution seperately. The model also ignores the temperature dependance of the plasma frequency due to the moderate range of heating experimentation in this work^45^. Figure 5(a) shows the relation of α witht the real and imaginary component of metal permitivitty. As α is increased, Re(ε) is decreased meaning a net reduction of metal conductivity and Im(ε) increases meaning a net increase of metal absorption. According to the power law of vibrational mode, we increase the linewidth (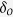) in Fig. 5(b) to mimic the temperature induced broadening. As 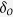 is increased, the transition slope of Re (n) decreases whereas the profile of Im (n) flattens meaning a net reduction of vibrational absorption. Figure 5(c) shows the reduction of the strength of the reflection peaks at R1, R2 and R4 regions as α is increased which is in agreement with the experimental results in Fig. 3 as the temperature is incrased. This is in agreement with the reduction of extinction efficiency as a function of increasing temperature observed in^43^.

**Figure 6:**
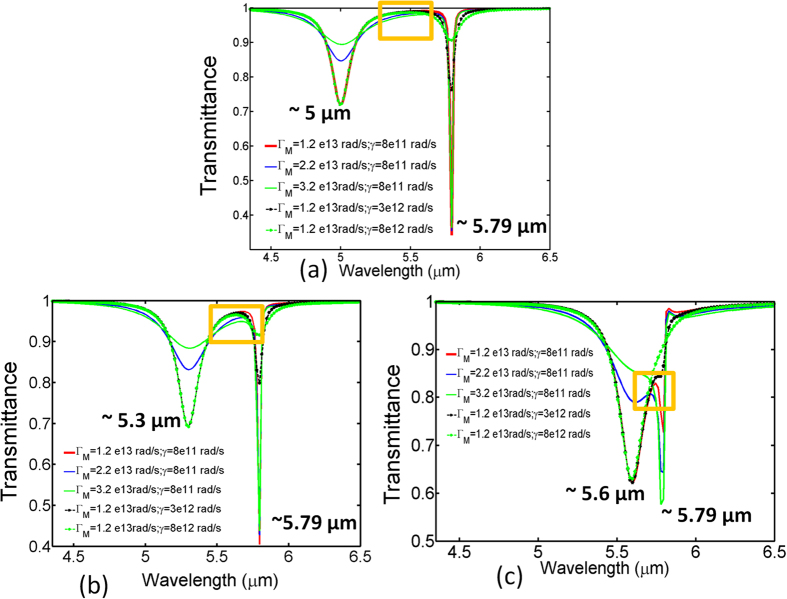
Simulated transmittance derived from the two particle model. “Bright mode” at (**a**) 5 μm (**b**) 5.3 μm (**c**) 5.6 μm. The orange rectangle indicates the EIT-like transmission window.

The overall resonance cotrast at the hybridized dip at R3 is also decreased primarily owing to the base line shift of the metal reflectivity on the blue side and the red side of the dip (Figure S8, supplemenary information). A close inspection will reveal further that the quality factor of the dip is also affected by the increase of α in agreement with the experimental results. However, it is still not clear how the temperature induced broadening of vibrational mode can affect the line shape of the hybridized mode. Hence, a more detailed interrogation is conducted in Fig. 6(d). It is obvious from the results that decrease of vibrational absorption with the increase of PMMA line width decreases the resonance contrast and quality factor of the hybridized mode. However, this time, the shift of the dip at R3 region is in the opposite direction implying a different mechanism. On the other hand, it has already been shown that resistive heating of the metal structures with the increase of α can shift the dip in the downward direction. Hence, the coupled system can possess a self-balanced mechanism through which the variation of the dip intensity can be compensated at elevated temperature. This is infact observed in Fig. 5(d) when α is increased after broadening of the vibrational mode occurs. The initial magnitude of the reflection dip at (α = 1, 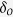 = 8e11 rad/s) is close to the final magnitude of the reflection dip when both metal structures and PMMA film are assumed to be at the elevated temperaute simultaneously (α = 1.8, 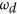 = 3e12 rad/s). However, throughout the mechanism, the quality factor of the dip is strongly affected with a net reduction of the average intensity I = (I_L_ + I_R_)/2. Althouth our current modeling results can slightly overestimate the actual thermal affect due to the lack of precise relationship of α in the nanostructure and *δ* of the PMMA thin film with temperature, the qualitative agreement will undoubtedly help further to devise a mechanism for reducing the relative intensity change of the coupled mode at high temperature. It is also not technically possible to set apart the contribution of resistive heating and vibrational broadening in the current experimental setup. Since preservation of large resonance contrast is particularly meaningful for smart on-chip microfluidic system where the mid IR loss of the aqueous solution needs to be sufficiently suppressed^46^, here we propose an approach to minimize the relative intensity change of the coupled mode dominant by the resistive heating of metal. Firstly, we suggest that the “bright mode” and the “quasi dark mode” needs to be sufficiently detuned from each other, which can be easily achieved by designing the nanostructures. Here, the nearby resonance at R2 is located around 5.3 μm in experiment after the index perturbation defined as below^47^,


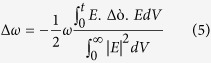


Here, E, 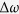, 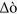
are the time averaged electric field, frequency shift from the perturbation theory, and change of dielectric constant, respectively. Secondly, the strength of the nearby resonance peak needs to be increased by surface current engineering of the metal loss while enhancing the spatial overlap with the vibrational mode so that the average resonance contrast at the dip is magnified. To support the former proposition, here we investigate into the two particle transmission model of a coupled system in the next section. while for the later, we consider a group of layouts and demonstrate the impact of the nearby resonance on the average intensity of the coupled dip.

### Two particle analysis

Zheludev *et al.* already reported 4 dB transmission change of the Fano resonance in the superconductor metamaterial as the temperature increases from 77 K to 200 K^48^. Here, we employ a two particle model of EIT-like transmission with arbitrary constants, where both the “bright mode” and “quasi dark mode” respond with the incoming light. The linear susceptibility (χ) of the coupled system can be expressed as below^49^,


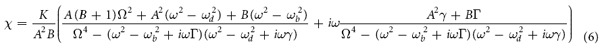


Here, (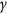, 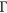) and (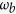) are the loss rate (line width) and resonance angular frequencies of the “quasi dark mode” and “bright mode”, respectively. 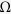 (8e13 rad/s) is the coupling strength and A (1) and B (0.25) are the dimensionless constants dictating the relative coupling of incoming radiation with the “bright mode” and “quasi dark mode”, respectively. K is the amplitude offset fixed at 3e26. The transmittance is calculated as 1-Im (χ) and plotted in Fig. 6. It is worthwhile to note that the change of transmission profile within the transparency window indicated by the orange rectangle is less pronounced as compared to the changes around the Eigen states indicated on the blue side (“bright mode”) and the red side (“quasi dark mode”) of the window when the loss rates are varied. Moreover, increasing the loss rate of the “bright mode” primarily reduces the transmission dip on the blue side whereas it occurs on the red side when the loss rate of the “quasi dark mode” is increased. However, the peak intensity in the window is increasingly affected as the “bright mode” wavelength approaches the “dark” mode wavelength located at 5.79 um even though the variation of the loss rates are kept constant. Note that the intensity peak in transmission is significantly correspondent to the intensity dip in reflection, i.e., R3 in our case. This clearly emphasizes on the requirement of detuning the “bright mode” and “dark mode” to some extent for suppressing the intensity change within the transmission window as the loss rates increase with temperature.

### Impact of Spatial Coupling on the Relative Intensity Change of the Hybridized Mode

**Figure 7:**
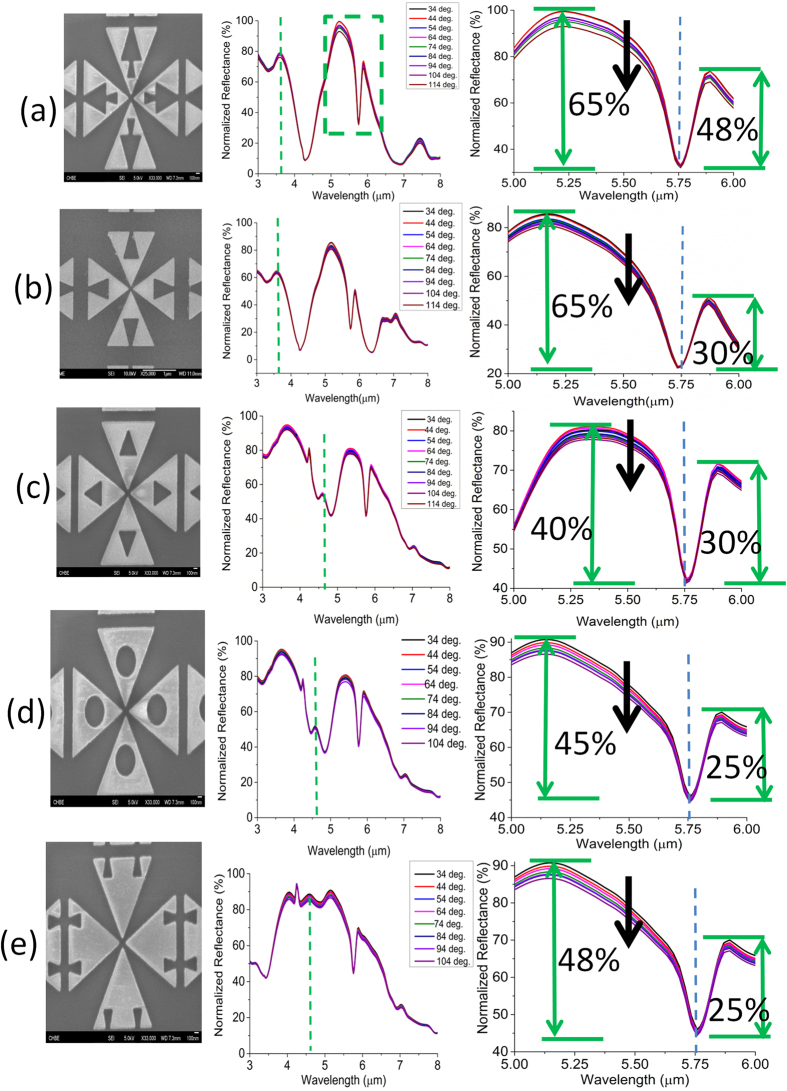
Role of spatial coupling on the hybridized mode in order to reduce the relative intensity change as the temperature is varied. Left panel: SEM image of case (**a–e**), mid panel: intensity change of the overall reflectance spectrum as a function of temperature, right panel: zoomed in change of the reflectance dip intensity at the hybridized mode as the temperature is varied. The green dashed rectangle is the hybridized reflectance dip under consideration. The green dashed line implies the longitudinal mode of the bow-tie triangle which is not the focus of this work (Figure S10, supplementary information).

Next, we focus on the role of the design of nanostructures on achieving large coupled resonance contrast and thereby decreasing the relative intensity change at high temperature. Note that the bow-tie arms of the proposed geometry are consisted of several voids which are the key determinants of its spatially and spectrally variant resonance characteristics. There are three specific objectives behind introducing voids into the solid pattern. Firstly, reducing the metal surface area will decrease the effect of damping, thereby will increase the quality factor of the resonance. Secondly, the voids can efficiently exploit the lighting rod effect of the corners of the triangular geometry and engineer the surface current for further enhancement of the dipolar moment^50^,^51^. Such enhancement can magnify the contrast of the nearby resonance as required for the improvement of the hybridized mode. Thirdly, the voids can increase the spatial coupling of the localized fields of the geometry with the PMMA overlayer and thus enhance the vibrational absorption (Figure S9, supplementary information). From the mechanical point of view, the voids will also mitigate the effect of large thermal expansion coefficient mismatch between the Au and PMMA layer. Here, the overall coupling coefficient (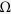) can be expressed as below^47^,


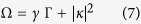


where 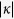 is the magnitude of the mutual coupling coefficient which can be engineered by the voids. Under strongly coupling assumption, it can be shown from equation 9 that 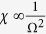, meaning no dependence of the EIT-like transmission on the loss rates^52^. The proposed geometry can be broken down into three different layouts based on the presence of the individual voids (left panel of Fig. 7(a,b and e)). Besides, we consider two more geometries in Fig. 7(c,d) with equivalent voids to justify the role of surface current engineering on the average contrast of the dip. It can be observed that the peak average contrast (56.5%) is achieved by the geometry in Fig. 7(a) which efficiently engineers the surface current at the dipolar mode and maximizes the spatial coupling with the thin film. The effect of temperature on the blue side and the red side of the dip, however, is nearly the same for different structures. We observe a larger intensity variation (5%) on the blue side of the dip. Thus, the percentage relative intensity change with respect to the initial average contrast reaches a minimum of 8.85%. On the other hand, the percentage change

increases to 14.3% in the structures of Fig. 6(c,d) which have rather lower average resonance contrast. Such increase is attributed to two factors: (i) reduced peak reflection on the both sides of the dip and (ii) increased reflection at the dip due to poor spatial coupling. Hence, the experimental results in Fig. 7 clearly signify the role of surface current engineering and spatial coupling on improving the robustness of the coupled resonance against harsh conditions for many challenging applications.

### Impact of Multiple Cycling on the Device Reliability

**Figure 8:**
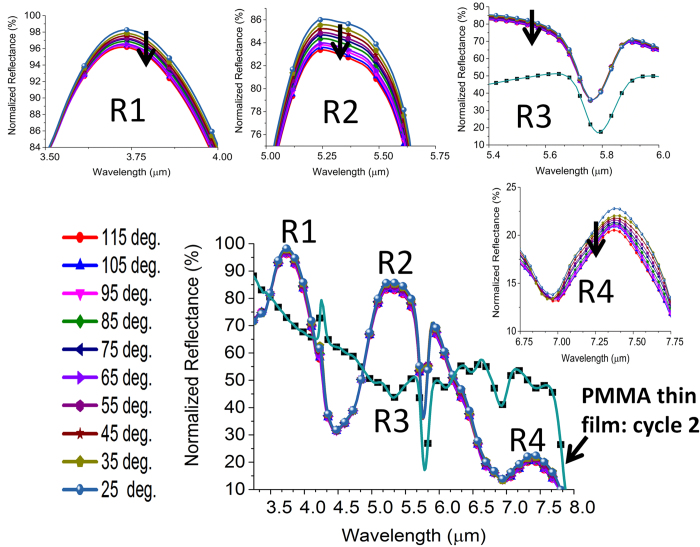
Intensity change with temperature at the important spectral positions (R1, R2, R3, R4) of the spectrum in cycle 3. Insets: zoomed in variations of intensity change at the spectral positions (R1, R2, R3, R4).

**Figure 9:**
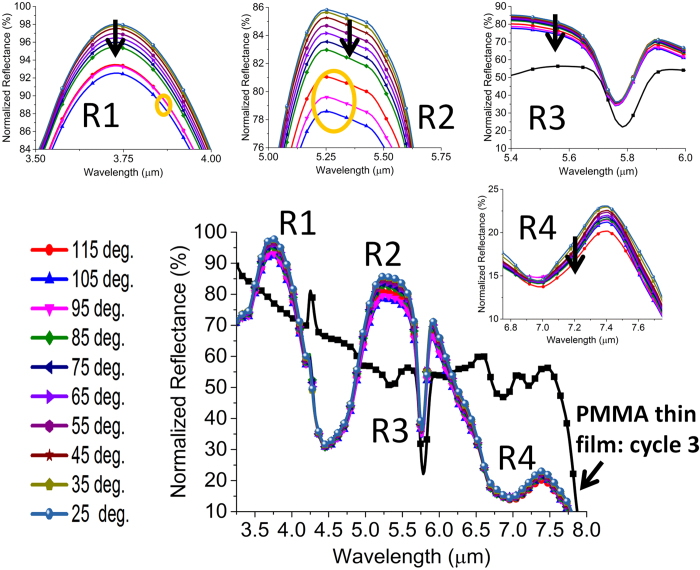
Intensity change with temperature at the important spectral positions (R1, R2, R3, R4) of the spectrum in cycle 4. Insets: zoomed in variations of intensity change at the spectral positions (R1, R2, R3, R4).

In this section, we interrogate the reliability of the composite device under multiple cycling and conclude that it is not the structural deformation of the nanopattterns but the modified surface chemistry between two chemically mismatched layers that affects the device performance. Thermal degradation can in fact impart deleterious effect on the reliability of polymer devices^53^. Especially, under alternate heating and cooling cycles, fatigue can generate in the polymer film and lead to device failure^54^. Hence, study of thin film degradation is technologically important for the new generation of hybrid plasmonic device. Although, PMMA has an excellent weatherability and scratch resistance, it has a very poor chemical resistance. However, owing to the range of temperature considered in this work, any chemical change of PMMA can be safely ignored in this work. On the contrary, thermal stress can build up due to the large mismatch of thermal expansion coefficient especially between Au and PMMA and PMMA and SiO_2_ and culminate in a fatigue over repeated cycles while weakening the adhesion bond. Here, the scenario can be optically probed using a multiband resonant structure which can be responsive to any localized change of adhesion over the repeated cycles. The results in Fig. 8 indeed depict the effect of thermal degradation in cycle 3. A significant change of reflectivity and resonance contrast are observed at the R2 and R4 regions meaning a peeling off of PMMA particularly from the large central cavity. The intensity and contrast of dipolar resonance at R2 and magnetic resonance at R4 strongly depend on the arrangement of the thin film within the cavity. We observe a decrease of intensity by at least 10% at the R2 region. A decrease of contrast by 20% and 10% is observed at the R2 and R4 region, respectively. This implies a global change of reflectivity in the spectral region defined by R2 and R4 eventually causing a net decrease of the resonance contrast of the dip at R3. The average contrast now becomes 45% as opposed to 56.5%. No change is observed in the R1 region meaning a reduced impact of adhesion weakening on the resonance having smaller spatial coverage. Under all circumstances of the third cycle, a regular trend between the absolute reflectivity and temperature is observed at the significant spectral positions (R1, R2, R3, R4), i.e., reflectivity drops as the temperature increases. However, this trend deviates in the fourth cycle when the stress developed becomes more detrimental for the reliability of the device. The results in R1 and R2 regions in Fig. 9 clearly indicate that the trend no longer holds in the range 95–115 deg. as marked by the orange circle. Such digression also has an influence on the intensity variation at R3. However, the trend is still observed at the R4 region which may collapse in the subsequent cycles. The results indicate the usefulness of the hybrid nanostructure with spectrally and spatially variant resonances for a more reliable monitoring of thermal status over repeated cycles. The failure condition can be easily identified by the sign of the differential change of intensity across the broad spectrum in many relevant applications.

**Figure 10:**
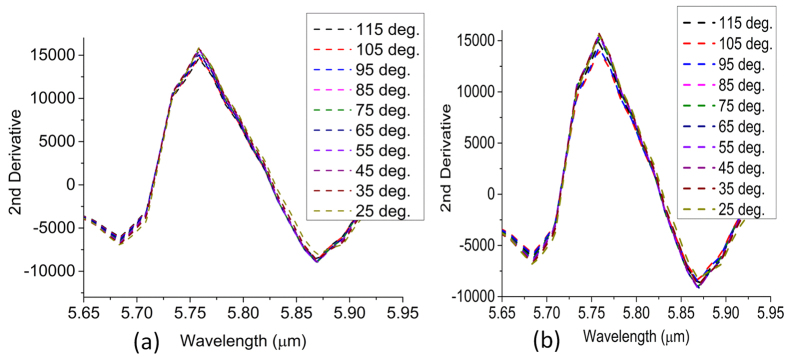
(**a**) Height AFM (**b**) phase AFM (**c**) probability histogram of the phase AFM images of the initial sample. (**d**) Height AFM (**e**) phase AFM (**f**) probability histogram of the phase AFM images after fourth cycle.

**Figure 11:**
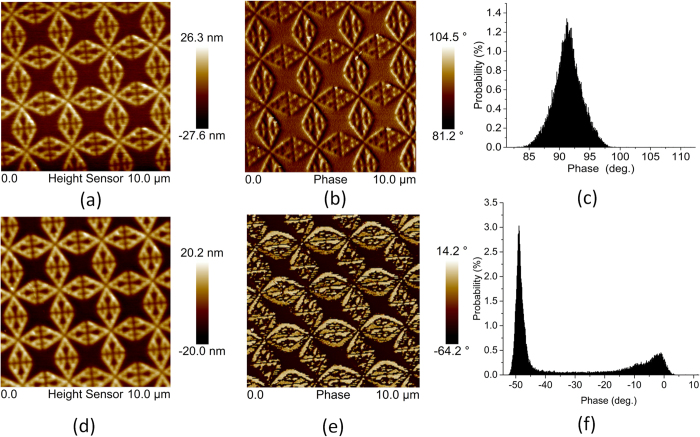
Variation of second derivative with temperature (**a**) after third cycle (**b**) after fourth cycle at R3 zone. The relative position of the black dashed line is altered in the fourth cycle as the stress is developed.

To further substantiate the findings on the premise of surface chemistry alternation, we employ the phase contrast AFM imaging to identify the the change of structural topography after cycle 4. It can be seen in Fig. 10 that conventional height AFM can not detect the change of topography due to the alternate heating and cooling in multiple cycles. Apparently, no distortion of the pattern is noticed in the AFM image as well. On the contrary, the phase images in Fig. 10(b,e) indicate a drastic change of topography as the thermal stress becomes significant in the fourth cycle. The probabilistic histograms in Fig. 10(c,f) also elucidate the difference between the initial sample and cycled sample. The profile in Fig. 10(f) implies that the topography change mainly occurs at the inner edge of the cavity where a complex interplay of the thermal stresses at Au-PMMA interface and PMMA-SiO_2_ interface can occur. Finally, the second derivatives of the reflectance dip at R3 are plotted in Fig. 11. Second derivative of the signal is a powerful tool for tracking minute change in the spectrum. Obviously, a change of slope is observed in Fig. 11(b) when the black dashed line visibly moves to a different relative position. This is a direct consequence of the deviation from the temperature-reflectance relation in the fourth cycle as discussed previously.

## Conclusion

In summary, we conduct a thermoplasmonic study of a triple band nanoantenna structure strongly coupled to a mid IR molecular mode for the first time. Localized heating of plasmonic nanostructures is of fundamental importance in translational research on thermoresponsive drug release and photothermal cancer therapy. Here, our study shows how such resistive heating of noble metal can be a concern for the aforementioned emerging applications of plasmonic device irrespective of the nature of the resonances. Unlike metamaterial absorber structures, here, we do not observe any resonance shift as the temperature of the device is increased as the coupling between the top metal and bottom metal reflector is absent^55^,^56^. However, we explore the relatively higher robustness of the coupled mode intensity against temperature increase in our experiment and perform numerical and analytical investigation into the phenomenon. Afterwards, we propose a mechanism based on mode detuning and surface current modulation for minimizing the relative intensity change of the hybrid mode. Finally, we perform the cycling test and identify the issue of adhesion weakening causing the deviation of global reflectance of the pattern and its temperature-reflectance relationship. Phase contrast AFM imaging further reveals the drastic topological change of the planar geometry due to the cycling effect and indicates the impact of surface chemistry rather than structural deformation on the reliability of the composite device. In particular, the spectrally and spatially variant multiple resonances of the nanostructure are found to be potentially useful for a deterministic thermal monitoring of localized change of surface chemistry between chemically mismatched materials. The average resonance contrast of the coupled dip still preserves itself to be stronger (45%) than the fresh thin film response (40%) under such thermal degradation. Hence, we believe that the proposed mechanism of enhancing coupled resonance contrast will be beneficial for the ultimate colorimetric detection in the presence of strong thermal degradation and resistive heating of plasmonic structures.

## Additional Information

**How to cite this article**: Hasan, D. *et al.* Thermoplasmonic Study of a Triple Band Optical Nanoantenna Strongly Coupled to Mid IR Molecular Mode. *Sci. Rep.* **6**, 22227; doi: 10.1038/srep22227 (2016).

## Supplementary Material

Supplementary Information

## References

[b1] Baffou G. & Quidant R. Thermo-plasmonics: using metallic nanostructures as nano-sources of heat. Laser & Photon. Rev. 7, 171–187 (2013).

[b2] Baffou G., Quidant R. & Girard C. Thermoplasmonics modeling: A Green’s function approach. Phys. Rev. B, 82 (2010). doi: http://dx.doi.org/10.1103/PhysRevB.82.165424.

[b3] Govorov Richardson & H. H. Generating heat with metal nanoparticles. Nano Today 2, 30 (2007).

[b4] Cao L., Barsic D. N., Guichard A. R. & Brongersma M. L. Plasmon-Assisted Local Temperature Control to Pattern Individual Semiconductor Nanowires and Carbon Nanotubes. Nano Lett. 7, 3523–3527 (2007). 17963415 10.1021/nl0722370

[b5] Li Z. H. & Truhlar D. G. Nanothermodynamics of metal nanoparticles. Chem. Sci. 5, 2605–2624 (2014).

[b6] Karker N., Dharmalingam G. & Carpenter M. A. Thermal Energy Harvesting Plasmonic Based Chemical Sensors. ACS Nano. 8, 10953–10962 (2014). 25280004 10.1021/nn504870b

[b7] Dregely D., Lindfors K., Lippitz M., Engheta N., Totzeck M. & Giessen H. Imaging and steering an optical wireless nanoantenna link. Nat. Comm. 5, 4354 (2014). doi: 10.1038/ncomms5354. PMC4102110 24993946

[b8] Gartia M. R. *et al.* Colorimetric Plasmon Resonance Imaging Using Nano Lycurgus Cup Arrays. Adv. Opt. Mat. 1, 68–76 (2013).

[b9] Hasan D., Ho C. P., Pitchappa P. & Lee C. Dipolar Resonance Enhancement and Magnetic Resonance in Cross-Coupled Bow-Tie Nanoantenna Array by Plasmonic Cavity. ACS Photon. 2, 890–898 (2015).

[b10] Xu X., Hasan D., Wang L. & Chakravarty S. Guided-mode-resonance-coupled plasmonic-active SiO_2_ nanotubes for surface enhanced Raman spectroscopy. Appl. Phys. Lett. 100, 191114 (2012). 22685345 10.1063/1.4714710 PMC3360636

[b11] Xu X., Li H., Hasan D., Ruoff R. S., Wang A. X. & Fan D. L. Near-Field Enhanced Plasmonic-Magnetic Bifunctional Nanotubes for Single Cell Bioanalysis. Adv. Funct. Mater. 23, 4332–4338 (2013).

[b12] Ayala-Orozco C., Urban C., Knight M. W., Urban A. S., Neumann O., Bishnoi S. W., Mukherjee S., *et al.* Au Nanomatryoshkas as Efficient Near-Infrared Photothermal Transducers for Cancer Treatment: Benchmarking against Nanoshells. ACS Nano. 8, 6372–6381 (2014). 24889266 10.1021/nn501871d PMC4076033

[b13] Poon L., Zandberg W., Hsiao D., Erno Z., Sen D., Gates B. D. & Branda N. R. Photothermal Release of Single-Stranded DNA from the Surface of Gold Nanoparticles Through Controlled Denaturating and Au-S Bond Breaking. ACS Nano. 4, 6395–6403 (2010). 20958080 10.1021/nn1016346

[b14] Alabastri A., Toma A., Malerba M., Angelis F. D. & Zaccaria R. P. High Temperature Nanoplasmonics: The Key Role of Nonlinear Effects ACS Photon. 2, 115–120 (2015).

[b15] Lee K.-L. & Wei P.-K. Enhancing Surface Plasmon Detection Using Ultrasmall Nanoslits and a Multispectral Integration Method. Small 6, 1900–1907 (2010). 20669239 10.1002/smll.201000598

[b16] Weis P., Garcia-Pomar J. L., Beigang R. & Rahm M. Hybridization Induced Transparency in composites of metamaterials and atomic media. Opt. Expr. 19, 23573–23580 (2011). 10.1364/OE.19.023573 22109237

[b17] Liu J., Xu G., Liu F., Kityk I., Liua X. & Zhena Z. Recent advances in polymer electro-optic modulators, RSC Adv. 5, 15784–15794 (2015).

[b18] Weeber J.-C., Hassan K., Saviot L., Dereux A., Boissière C., Durupthy O., Chaneac C., Burov E. & Pastouret A. Efficient photo-thermal activation of gold nanoparticle-doped polymer plasmonic switches. Opt. Expr. 20, 27636–27649 (2012). 10.1364/OE.20.027636 23262712

[b19] Nugen S. R., Asiello P. J., Connelly J. T. & Baeumner A. J. PMMA biosensor for nucleic acids with integrated mixer and electrochemical detection. Biosens. and Bioelectron 24, 2428–2433 (2009). 10.1016/j.bios.2008.12.025 19168346

[b20] Gholipour B., Zhang J., MacDonald K. F., Hewak D. W. & Zheludev N. I. An All-Optical, Non-volatile, Bidirectional, Phase-Change Meta-Switch. Adv. Mater. 25, 3050–3054 (2013). 23625824 10.1002/adma.201300588

[b21] Kaminský J., Bour P. & Kubelka J. Simulations of the Temperature Dependence of Amide I Vibration. Journal of Phys. Chem. A. 115, 30–34 (2011). 21141980 10.1021/jp1084839

[b22] Falconer K. Fractal Geometry: Mathematical Foundations and Applications. John Wiley & Sons, Ltd. 2, 27–38 (2005).

[b23] Aouani H. *et al.* Plasmonic Nanoantennas for Multispectral Surface-Enhanced Spectroscopies. Journal of Phys. Chem. C. 117, 18620–18626 (2013).

[b24] Eter A. E. *et al.* Huge light-enhancement by coupling a bowtie nano-antenna’s Plasmonic resonance to a photonic crystal mode. Opt. Expr. 22, 14464–14472 (2014). 10.1364/OE.22.014464 24977543

[b25] Gramotnev D. K. & Bozhevolnyi S. I. Nanofocusing of electromagnetic radiation, Nat. Photon. 8, 13–22 (2014).

[b26] Mock J. J., Smith D. R. & Schultz S. Local Refractive Index Dependence of Plasmon Resonance Spectra from Individual Nanoparticles, Nano Lett. 3, 485–491 (2003).

[b27] Niklasson G. A., Granqvist C. G. & Hunderi O. Effective medium models for the optical properties of inhomogeneous materials. Appl. Opt. 20, 26–30 (1981). 20309062 10.1364/AO.20.000026

[b28] Lahiri B., McMeekin S. G., De La Rue R. M. & Johnson N. P. Enhanced Fano resonance of organic material films deposited on arrays of asymmetric split-ring resonators (A-SRRs). Opt. Expr. 21, 9343–9352 (2013). 10.1364/OE.21.009343 23609645

[b29] Qian J. *et al.* Effect of Edge Rounding on the Extinction Properties of Hollow Metal Nanoparticles. Plasmonics. 8, 955–962 (2013).

[b30] Raziman T. V. & Martin O. J. F. Polarisation charges and scattering behaviour of realistically rounded plasmonic nanostructures. Opt. Expr. 21, 21500–21507 (2013). 10.1364/OE.21.021500 24104025

[b31] lbella P. *et al.* Experimental Verification of the Spectral Shift between Near- and Far-Field Peak Intensities of Plasmonic Infrared Nanoantennas. Phys. Rev. Lett. 110, 203902 (2013). 25167410 10.1103/PhysRevLett.110.203902

[b32] Zuloaga J. & Nordlander P. On the Energy Shift between Near-Field and Far-Field Peak Intensities in Localized Plasmon Systems. Nano Lett. 11, 1280–83 (2011). 21319841 10.1021/nl1043242

[b33] Menzel C. *et al.* The spectral shift between near- and far-field resonances of optical nano-antennas. Opt. Expr. 22, 9971–9982 (2014). 10.1364/OE.22.009971 24787879

[b34] Fryer D. S. *et al.* Dependence of the Glass Transition Temperature of Polymer Films on Interfacial Energy and Thickness. Macromolecules. 34, 5627–5634 (2001).

[b35] Taubert R., Hentschel M, Kästel J. & Giessen H. Classical Analog of Electromagnetically Induced Absorption in Plasmonics. Nano Lett. 12, 1367–1371 (2012). 22273467 10.1021/nl2039748

[b36] Tokmakoffa A. & Fayer M. D. Homogeneous vibrational dynamics and inhomogeneous broadening in glass-forming liquids: Infrared photon echo experiments from room temperature to 10 K. J. Chem. Phys. 103, 2810–2826 (1995).

[b37] Zhang Z., Zhao P., Lin P. & Sun F. Thermo-optic coefficients of polymers for optical waveguide applications. Polymer. 47, 4893–4896 (2006).

[b38] Villetti M. A. *et al.* Thermal degradation of natural polymers. Journal of Therm. Anal. and Cal. 67, 295–303 (2002).

[b39] Shin H. S. *et al.* Glass Transition Temperature and Conformational Changes of Poly(methyl methacrylate) Thin Films Determined by a Two-Dimensional Map Representation of Temperature-Dependent Reflection-Absorption FTIR Spectrum. Langmuir. 18, 5953–5958 (2002).

[b40] Hasan D. & Alam M. S. Ultra-Broadband Confinement in Deep Sub-Wavelength Air Hole of a Suspended Core Fiber. IEEE J. Lightw. Tech. 32, 1434–1441 (2014).

[b41] Anton K. *et al.* Influence of surface oxidation on plasmon resonance in monolayer of gold and silver nanoparticlesKuzma. J. of App. Phys. 112, 103531 (2012).

[b42] Shukla R. *et al.* Biocompatibility of Gold Nanoparticles and Their Endocytotic Fate Inside the Cellular Compartment: A Microscopic Overview. Langmuir. 21, 10644–10654 (2005). 16262332 10.1021/la0513712

[b43] Bosman M. *et al.* Encapsulated Annealing: Enhancing the Plasmon Quality Factor in Lithographically–Defined Nanostructures. Scient. Rep. 4, 5537 (2014). 10.1038/srep05537 PMC4078311 24986023

[b44] Alabastri A. *et al.* High Temperature Nanoplasmonics: The Key Role of Nonlinear Effects. ACS Photon. 2, 115–120 (2015).

[b45] Virk M. *et al.* Thermal Plasmonic Sensor Platform: Resistive Heating of Nanohole Arrays. Nano Lett. 14, 3544–3549 (2014). 24807397 10.1021/nl5011542

[b46] Adato R. & Altug H. *In-situ* ultra-sensitive infrared absorption spectroscopy of biomolecule interactions in real time with plasmonic nanoantennas. Nat. Comm. 4, 2154 (2014). doi: 10.1038/ncomms3154 PMC3759039 23877168

[b47] Alici Gallardo, I. F. Detecting secondary structure and surface orientation of helical peptide monolayers from resonant hybridization signals. Scient. Rep. 3, 2956 (2013). 10.1038/srep02956 PMC3797430 24129763

[b48] Fedotov V. A. *et al.* Temperature control of Fano resonances and transmission in superconducting metamaterials. Opt. Expr. 18, 9015–9019 (2010). 10.1364/OE.18.009015 20588747

[b49] Manukumara M. *et al.* Tailoring the slow light behavior in terahertz metasurfaces. App. Phys. Lett. 106, 181101 (2015).

[b50] Shafiei F. *et al.* A subwavelength plasmonic metamolecule exhibiting magnetic-based optical Fano resonance. Nat. Nanotech. 8, 95–100 (2013). 10.1038/nnano.2012.249 23353675

[b51] Gramotnev D. K. & Bozhevolnyi S. I. Nanofocusing of electromagnetic radiation, Nat. Photon. 8, 13–22 (2014).

[b52] Lu C. *et al.* An actively ultrafast tunable giant slow-light effect in ultrathin nonlinear metasurfaces. Light: Sci. & Appl. 4, e302 (2015).

[b53] Cheneler D. & Bowenb J. Degradation of polymer films. Soft Mat. 9, 344–358 (2013).

[b54] Singh B. & Sharma N. Mechanistic implications of plastic degradation, Pol. Degrad. and Stab. 93, 561–584 (2008).

[b55] Corey S. *et al.* Stable high temperature metamaterial emitters for thermophotovoltaic applications, Appl. Phys. Lett. 104, 201113 (2014).

[b56] Woolf D. *et al.* Heterogeneous metasurface for high temperature selective emission, Appl. Phys. Lett. 105, 081110 (2014).

