# Supplementary material for: Thermoplasmonic Study of a Triple Band Optical Nanoantenna Strongly Coupled to Mid IR Molecular Mode
Source: Sci Rep. 2016 Feb 26;6:22227. doi: 10.1038/srep22227 (PMC4768094; doi:10.1038/srep22227)
Supplement: Supplementary Information [file srep22227-s1.pdf]

Thermoplasmonic Study of a Triple Band Optical Nanoantenna Strongly Coupled to Mid IR Molecular Mode

Dihan Hasan[1,2,3,4], Chong Pei Ho[1,2,4], Prakash Pitchappa[1,2,4], Bin Yang[3*], Chunsheng Yang[3],

and Chengkuo Lee[1,2,4]

[1]*Department of Electrical & Computer Engineering, National University of Singapore, 4 Engineering Drive 3, 117576, Singapore*

[2]*Center for Intelligent Sensors and MEMS, National University of Singapore, 4 Engineering Drive 3, Singapore 117576*

[3]*National Key Laboratory of Science and Technology on Micro/Nano Fabrication, Department of Micro/Nano Electronics, Shanghai Jiao Tong University, Dong Chuan Road 800, 200240 Shanghai, P. R. China.*

[4]*NUS Suzhou Research Institute (NUSRI), Suzhou Industrial Park, Suzhou, P. R. China 215123*

Corresponding Author: Bin Yang* and Chengkuo Lee[&]

***E-mail: binyang@sjtu.edu.cn;**
[&*]E-mail: elelc@nus.edu.sg

Supplementary information

Setup for thermal experiment

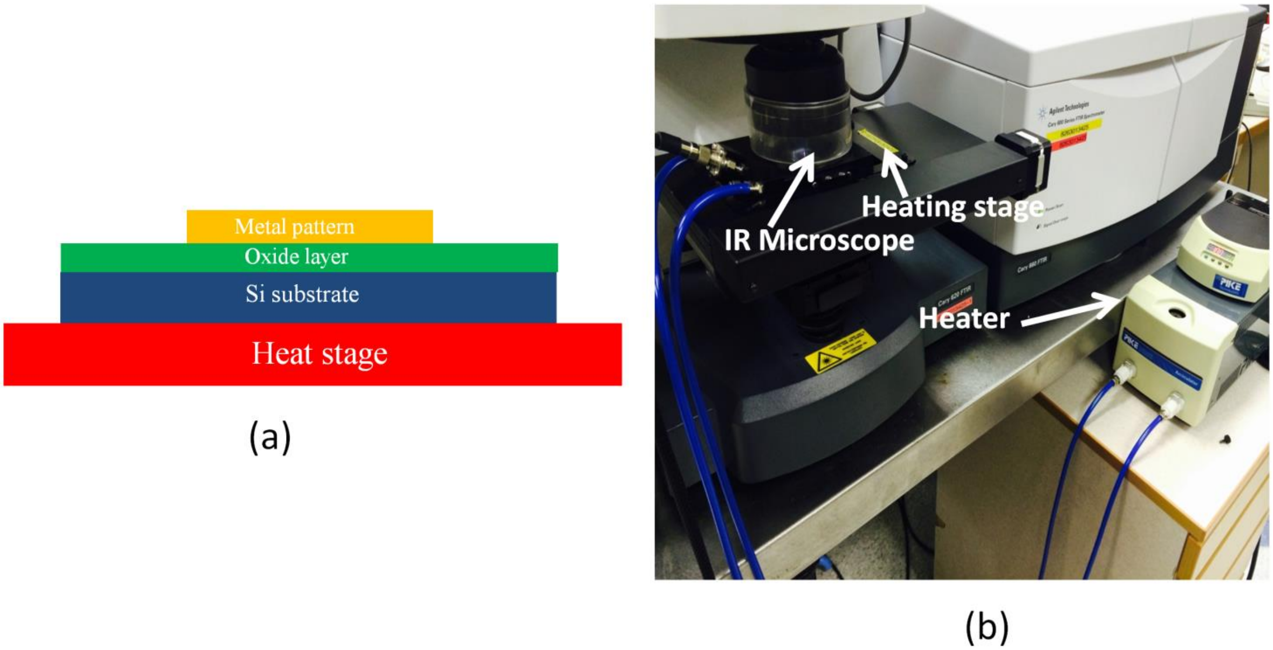

Fig. S1 (a) Schematic of the setup (b) the IR microscope coupled heating stage for thermal study

<u>Corner roundedness of the structure:</u>

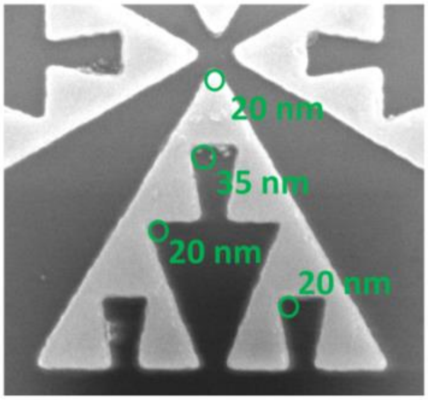

Fig. S2 Corner roundedness of the nanoantenna structure

<u>Matching the asymmetric reflection dip by varying the vibrational mode wavelength:</u>

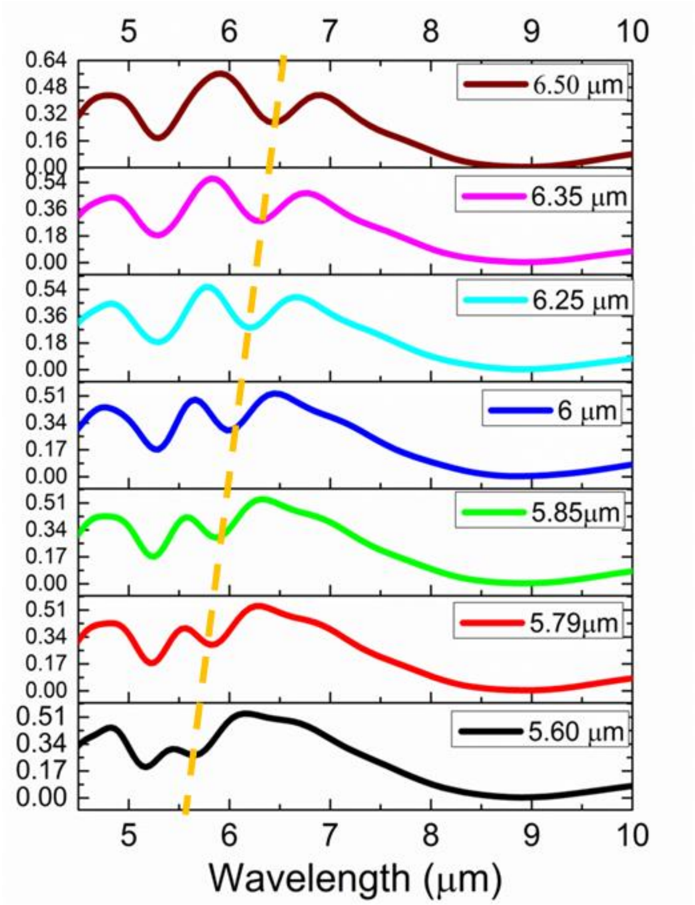

Fig. S3 Change of line shape of the coupled mode as the vibrational wavelength is red shifted

Background reflection from the substrate:

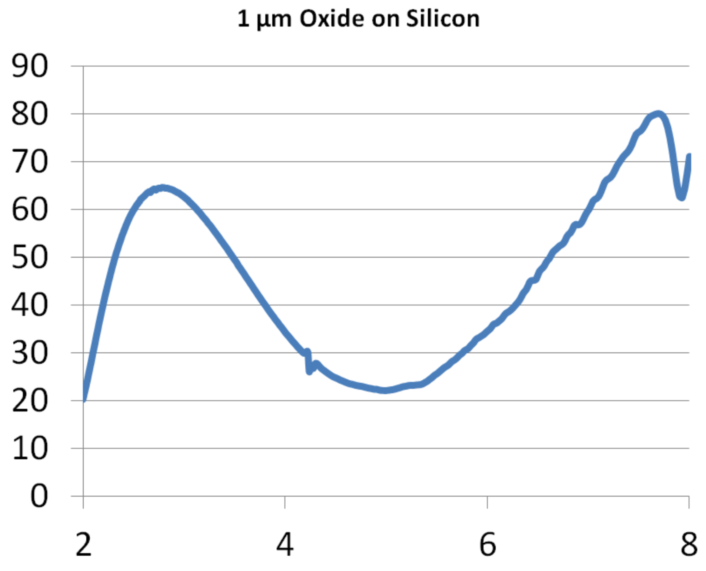

Fig. S4 Reflection peaks of the substrate

Experimental transmission peak and reflection dip in the EIT window

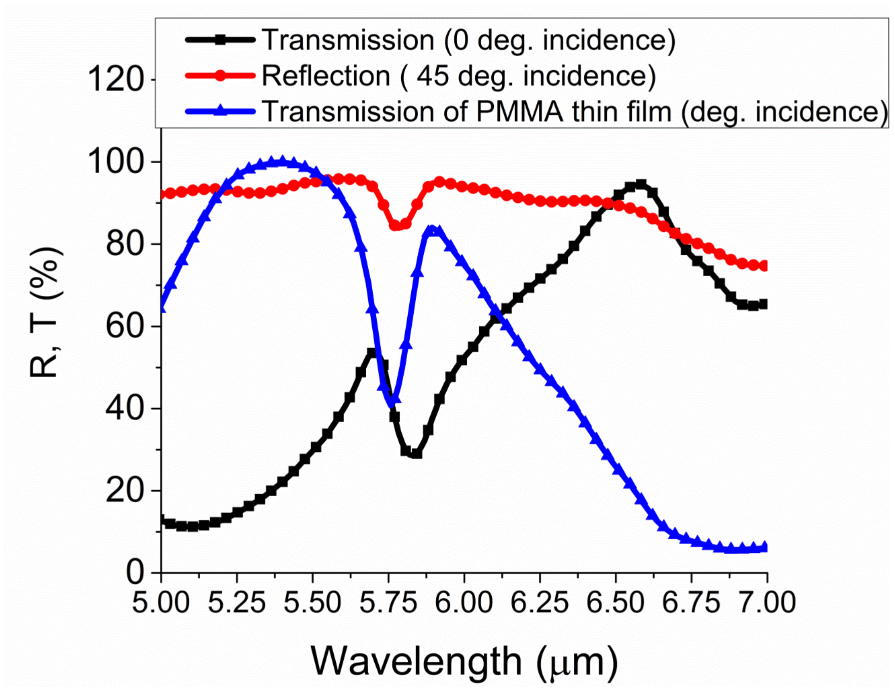

Fig. S5 Experimentally observed transmission and reflection in the EIT window

Effect of real part of PMMA refractive index change

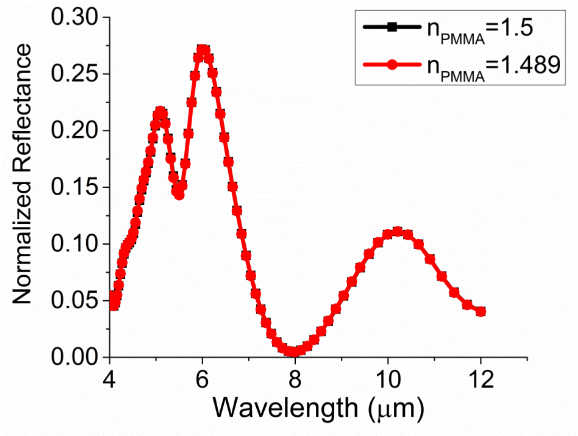

Fig. S6 Effect of change of Re(n) of PMMA as the temperature is increased by 100 deg.

Thermal stress study

The thermal distribution of the structure is obtained by numerically solving the heat diffusion equation simplified for heat conduction as below,

$$-\Delta.(\mathrm{k}\,\Delta T) = \mathrm{Q}$$

Here, k is the thermal conductivity and Q is the heat flux. The heating temperature and the reference temperature is fixed at 110 deg. and 25 deg., respectively. Thermal stress of the of the patterns is modelled by finite element method (FEM) deploying the following sets of equations ,

$$-\Delta\sigma = -\Delta.\left(\begin{bmatrix}\epsilon_x\\ \epsilon_y\\ \gamma_{xy}\end{bmatrix} - \begin{bmatrix}\alpha\\ \alpha\\ 0\end{bmatrix}(1+\nu)(\mathrm{T}-\mathrm{T}_{ref})\right) = F$$

$$\sigma = D\epsilon$$

$$\sigma_v = \sqrt{\frac{\sigma_x^2+\sigma_y^2+(\sigma_x-\sigma_y)^2}{2}}$$

Here σ is the stress tensor, $\varepsilon_{x/y}$ are the normal strain components, γ_{xy} is the shear strain component, D is the elasticity matrix of an isotropic material consists of Young's modulus E and Poisson's ratio ν, α is the thermal expansion coefficient, T the heating temperature (110˚C) and T_{ref} the reference temperature (25˚C). External force F is set to zero and longitudinal strain component is neglected in the thermal stress analysis. Von Mises stress (σ_v) is observed for the quantitative study of the thermal stress whose normal components are σ_x and σ_y.Table I lists the relevant mechanical parametes for the simulation.

Table I: Mechanical properties of different materials

Material	Melting Point (°C)	CTE (α [$10^{-6}K^{-1}$])	Young's Modulus (E [GPa])	Poisson ratio (μ)	Thermal Conductivity (K [W/m.k])
Gold	1,064	14	79	0.43	318
Chromium	1,907	4.9	279	0.21	93.9
PMMA	160	70	1.8-3.1	0.4	0.17-.0.25
SiO_2	1600	0.5	70	0.17	1.4

Fig.7 (a),(b) and (c) show the thermal distribution obtained by the heat conduction simulation. Fig. 7(d) shows the Von Mises stress distribution without the PMMA top layer. It can be seen that, the stress is highly localized at the sharp corners of the geometry. Fig. 7(e) shows the case when the PMMA overlayer is present. Due to the low stress of PMMA, a stress relaxation effect is observed especially at the corners when the layer is included [1]. The deformation plot also does not show any change of dimension due to the interplay of the compressive and tensile stress. Neverthless, the magnitude of the Von Mises stress at the mid plane of the pattern is still comparable to the case when the overlayer is absent.

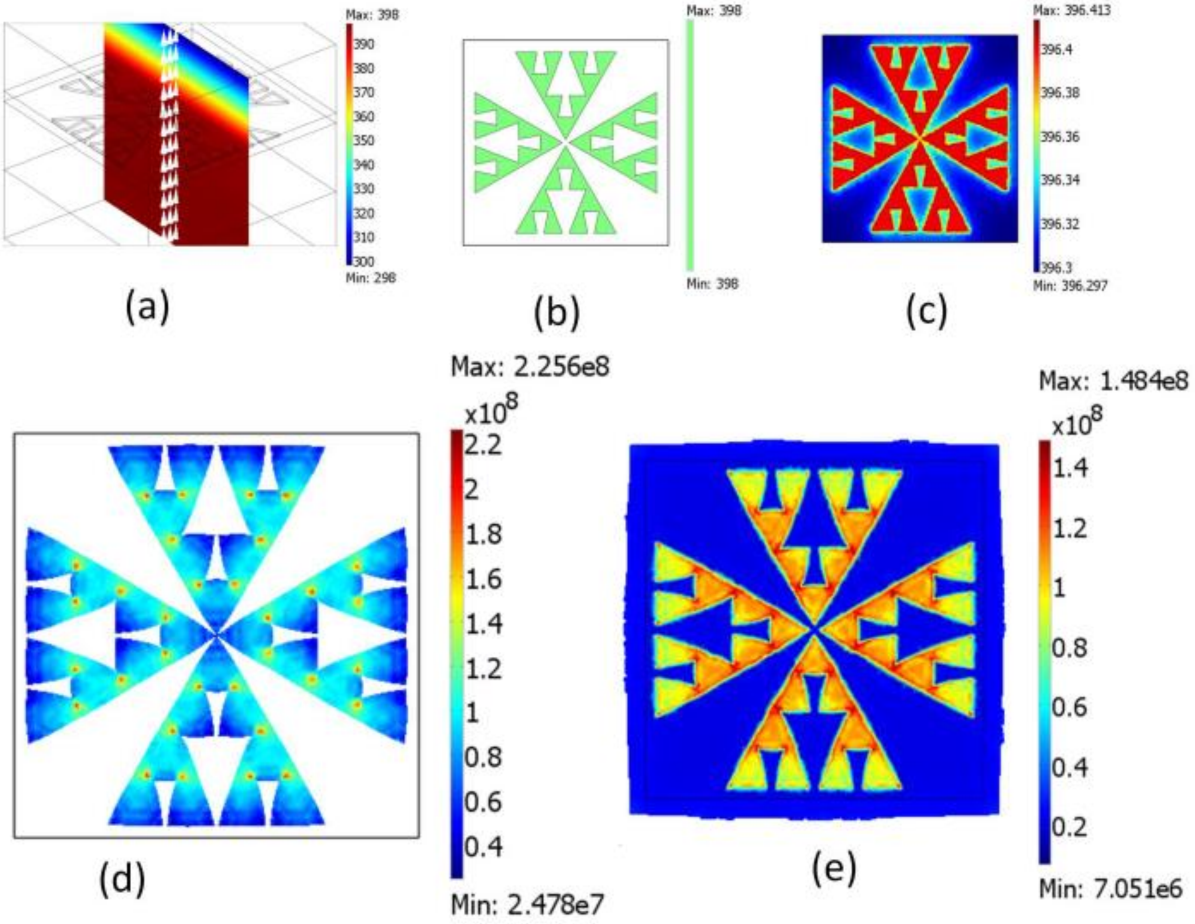

Fig. S7 (a) Heat flux distribution (b) thermal distribution without the PMMA overlayer (c) thermal distribution with the PMMA overlayer. Von Mises stress distribution at the mid plane of the pattern (d) without the PMMA overlayer (e) with the PMMA overlayer obtained by the FEM simulation

[1] Md. Faruque Hossain, Hau Ping Chan, Abbas Z. Kouzani, and Md. Osman Goni, "Generalized characteristics of photo-elastic birefringence in polymer strip waveguides," Opt. Mater. Express 5, 1030-1044 (2015)

Effect of increasing temperature on the reflectivity of metal thin film: simulation and experiment

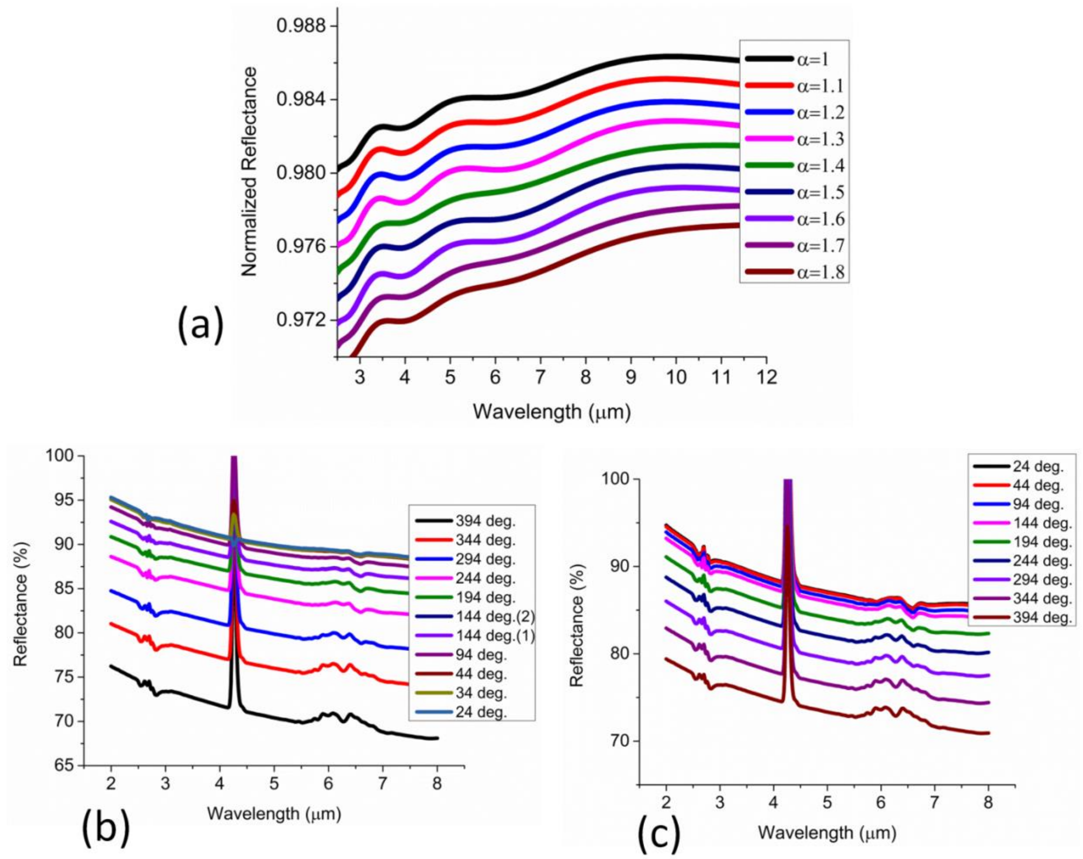

Fig. S8 (a) Effect of α on the reflectance of a 100 nm thin gold film on oxide substrate (b) experimental reflectivity change in the forward temperature cycling test (c) experimental reflectivity change in the backward cycling test

Effect of plasmonic voids on increasing the spatial overlap of the coupled mode

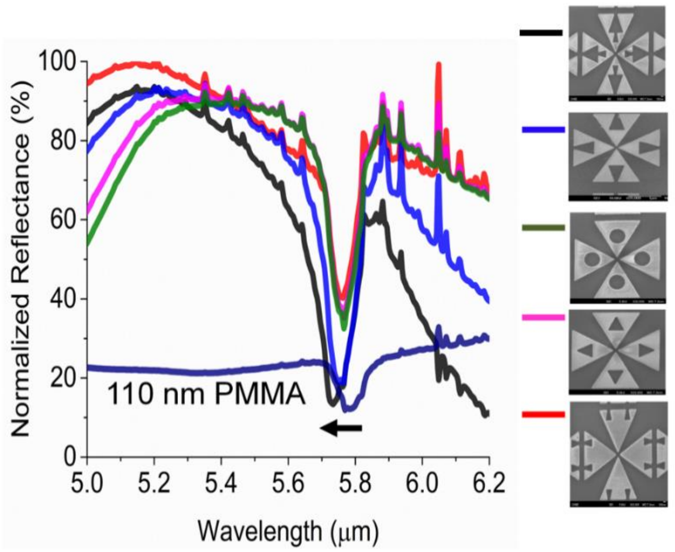

Fig. S9 Effect of nanostructure voids on the coupling of plasmonic mode with the PMMA thin film resonance mode

Origin of longitudinal mode in bow tie nanostructure in a polarization independent configuration

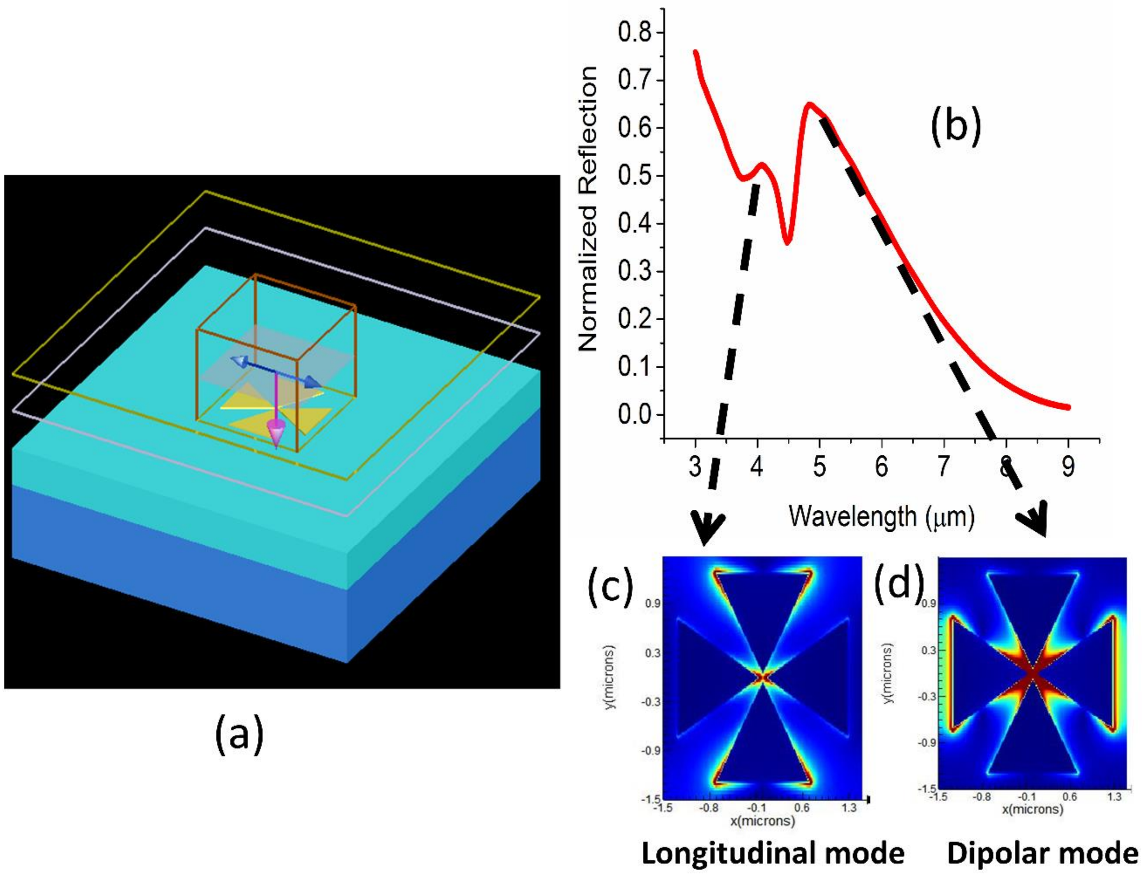

Fig. S10 Spectral and spatial characteristics of the dipolar mode and longitudinal mode of polarization independent bow tie nanostructure. (a) 3D schematic of the bow tie (b) normalized reflection spectrum (c) E- field distribution of the longitudinal mode (d)E-field distribution of the dipolar mode
